# Associations between constipation risk and lifestyle, medication use, and affective symptoms in patients with schizophrenia: a multicenter cross-sectional study

**DOI:** 10.1007/s00127-024-02729-8

**Published:** 2024-07-20

**Authors:** Che-Yu Chiang, Su-Chen Lo, Jason W. Beckstead, Chiu-Yueh Yang

**Affiliations:** 1 Department of Family and Community Medicine, Cathay General Hospital, Taipei, Taiwan; 2 National Yang Ming Chiao Tung University, Taipei, Taiwan; 3 College of Public Health, University of South Florida, Tampa, FL USA

**Keywords:** Anxiety symptoms, Constipation, Depressive symptoms, Predictors, Schizophrenia

## Abstract

**Purpose:**

To investigate the association between lifestyle and atypical antipsychotic drug use in patients with schizophrenia and the risk of constipation and to assess the impact of anxiety and depressive symptoms on constipation risk.

**Methods:**

Cross-sectional convenience sampling was employed, and 271 participants aged 20–65 were enrolled. Data were collected via a structured questionnaire comprising participants’ demographic data, medication information, dietary behavior assessment, and the Baecke Physical Activity Questionnaire, Beck Depression Inventory-II, and Beck Anxiety Inventory. IBM SPSS 24.0 with multivariate logistic regression was used for data analysis. We performed a subgroup analysis of anticholinergic drugs via multivariate logistic regression.

**Results:**

In total, 180 participants had functional constipation; risk factors included female sex, anxiety symptoms, depressive symptoms, and quetiapine and aripiprazole use. Patients who drank more than 3,000 cc of water daily or used risperidone were less likely to have functional constipation. Depressive and anxiety symptoms were risk factors even after adjusting for sex, use of anticholinergics and laxatives, consuming two servings of fruit, consuming three servings of vegetables, consuming more than 3,000 cc of water daily, physical activity, medical comorbidity, chlorpromazine equivalent dose, and atypical antipsychotic use. Similar associations were found for two affective symptoms and functional constipation in the subgroup analysis of anticholinergic drugs.

**Conclusion:**

The prevalence of functional constipation in patients with schizophrenia was 66.4%. The risk factors included female sex, anticholinergics, aripiprazole, quetiapine, and depressive and anxiety symptoms. Risperidone users and those who drank 3000 cc of water daily were less likely to have constipation.

## Introduction

Constipation significantly affects quality of life [1] and sleep, leading to potential bowel obstruction and increased mortality [2]. Its prevalence is 15.3% in the general population [3] but is much greater among patients with schizophrenia—over 30% in northern Europe [4] and 59.8% in Taiwan [5]. This heightened risk is linked to the side effects of antipsychotics, sedentary lifestyles, and the use of anticholinergics to mitigate these effects [6]. This elevated risk is largely attributed to the side effects of antipsychotics, particularly clozapine and olanzapine, which impair gastrointestinal motility [7, 8]. Furthermore, lifestyle factors such as reduced physical activity and unhealthy dietary behavior, which are common in patients with schizophrenia, exacerbate this issue [9, 10].

Constipation is more prevalent in Taiwan than in Western countries and is largely influenced by the use of antipsychotic medications such as clozapine and olanzapine, which impede gastrointestinal motility. National Health Insurance in Taiwan, which makes healthcare more affordable, may contribute to increased use of these drugs. Lifestyle factors, such as lower physical activity levels [9] and higher rates of overweight individuals among the schizophrenia population in Taiwan [9, 10], also heighten constipation risk [11]. Additionally, the gut microbiota, which affects metabolism and appetite, plays a role, although the importance of dietary fiber and water intake is often overlooked in studies.

While previous research has noted the high prevalence of constipation in schizophrenia patients, it often ignores the combined impact of medication and lifestyle factors. This study addresses these factors, offering a comprehensive analysis crucial for developing targeted strategies.

### Background

#### Constipation is associated with various risk factors

Demographically, female and urban populations are more susceptible to constipation. Lifestyle risk factors include insufficient intake of fiber and water and physical inactivity [12]. Concerning health conditions, along with organic diseases [12, 13], anxious and depressed moods worsen constipation [12]. Two northern European surveys demonstrated that clozapine treatment increases the risk of constipation in patients with schizophrenia [4, 14]. Likewise, second-generation antipsychotic medications, such as those targeting dopamine 2 and serotonin 2A receptors, as well as antidepressants and anticholinergics, can increase the risk of constipation in individuals with schizophrenia [5, 15, 16]. Compared with patients taking risperidone, patients taking clozapine are 3.97 times more likely to require laxatives, while those taking other atypical psychotics have a 1.70-–2.27-fold greater risk of constipation [8]. Anticholinergics are utilized to alleviate the side effects of antipsychotics, and some antidepressants and first-generation low-potency antipsychotics with anticholinergic effects can cause constipation [13, 15]. In addition, the interactions between anticholinergics and clozapine or quetiapine can increase the risk of constipation [7].

Previous studies have indicated that individuals with schizophrenia engage in less physical activity than the general population [9, 17], with those in Taiwanese rehabilitation facilities being less active than their counterparts in Belgium [9]. Their diet typically includes fewer vegetables and fruits, which does not lead to constipation. Research also suggests a weak link between inadequate water intake and constipation [12]. A study in eastern Taiwan revealed that patients with schizophrenia taking atypical antipsychotics had a 3.48-fold increased risk of constipation, and those taking anticholinergics had a 1.08-fold increased risk [5]. However, physical activity, a high-fiber diet, and demographic characteristics were not significantly associated with constipation in this population [5].

In this study, we analyzed how demographic factors, medication use, water intake, diet, physical activity, and anxiety and depressive symptoms correlate with constipation in schizophrenia patients. The aims of this study were as follows:

1. Explore how patients’ lifestyles affect constipation risk
2. Assess constipation risks associated with atypical antipsychotic use
3. Examine the impact of anxiety and depressive symptoms on constipation.

## Methods

### Research design

This cross-sectional study across ten rehabilitation units in two psychiatric hospitals investigated the impact of factors such as sex, anticholinergic and second-generation antipsychotic usage, fiber and water intake, and affective symptoms on functional constipation. Although 50.92% of participants (*n* = 138) still experienced constipation with laxative use, laxatives were included as a control variable in our regression model to assess their effect.

### Participants and setting

Participants were selected from ten hospital-based rehabilitation institutions in northern Taiwan. These individuals had chronic and persistent conditions but were capable of regularly engaging in therapeutic activities. The research subjects could participate in rehabilitation activities and functional rehabilitation tasks in the hospital. With prior approval, patients could also independently engage in activities and shopping outside the hospital. The inclusion criteria included the following:

1. Patients diagnosed with schizophrenia.
2. Patients aged 20–65 were recruited from daycare centers or chronic wards in the included hospitals.
3. Patients who regularly used antipsychotics for more than 10 weeks.
4. After providing an explanation, patients could clearly express themselves and sign the consent form for participation in this study.

Patients with cognitive impairment, intellectual disturbance, or substance use disorders were excluded.

### Measures

The data collected included demographic characteristics, water intake, vegetable intake, clinical characteristics, medication information, physical activity level, and depressive and anxiety symptoms.

#### Demographic data and lifestyle factors

Data on sex, age (years), duration of disease (years), occupational training status, and body mass index (kg/m^2^) were collected. Lifestyle factors, such as smoking status, drinking status, daily fluid intake, daily fruit intake, and daily vegetable intake, were associated with constipation. Due to the hospital-based rehabilitation setting of this study being located in a mountainous area, we adopted the definition of drinkers proposed by Chu et al. [18], which states that individuals who have consumed alcohol at least once in the past year are classified as drinkers.

#### Clinical characteristics and medication information

The clinical characteristics included disease duration, medical comorbidities, laxative use, anticholinergic anti-Parkinson agents (benztropine, biperiden, trihexyphenidyl, and amandine), sedative-hypnotic drugs, antidepressants, and antipsychotics. The imipramine 50 mg equivalent dose was computed [19]. This study obtained the medication histories from patients’ medical records. The first-generation antipsychotics include chlorpromazine, clotiapine, haloperidol, flupentixol, sulpiride, trifluoperazine, and fluphenazine decanoate. The chlorpromazine 100 mg equivalent dose was computed to calculate the relative antipsychotic dose [20], and the doses of sedative-hypnotic drugs were computed using the equivalent benzodiazepine calculator based on the diazepam 10 mg equivalent dose [19]. Calculating equivalent dosages of medications involves verifying from medical records that there has been no change in medication for more than ten weeks and recording the drug information using a structured questionnaire during the data collection phase. Medical comorbidities, including hypertension, diabetes, heart disease, cancer, thyroid disease, and pulmonary disease, are defined as chronic conditions that currently require treatment to assist in stabilization. In our study, if the participants took two atypical antipsychotics, we examined the relationship between each atypical antipsychotic and functional constipation separately.

#### Physical activity level

The Baecke physical activity questionnaire (BPAQ) assesses self-reported physical activity levels during work, leisure, and sports, focusing on frequency and duration [21]. It has been utilized internationally, including by Belgian researchers studying schizophrenia patients^22^, and was adapted in Hong Kong to include a housework index^23^. Its application in Taiwan confirmed its high reliability and validity through factor analysis, achieving a KMO value of 0.925 and internal consistency of 0.875 [9].

#### Depressive symptoms

Depressive symptoms were evaluated using the Beck Depression Inventory-II (BDI-II) [24], a 21-item self-report questionnaire on a 4-point scale (0–3), with scores ranging from 0–63 indicating severity. The BDI-II has high internal consistency (0.89–0.90) and criterion validity with the Hamilton Depression Rating Scale (0.71). The traditional Chinese version showed an internal consistency of 0.94 and a split-half reliability of 0.91. A score of ≥ 17 on the BDI-II suggests depressive symptoms [25].

#### Anxiety symptoms

The Beck Anxiety Inventory (BAI) [26], a 21-item scale (0–3) for assessing anxiety in individuals aged 17 years and older, aligns with the DSM-III and DSM-III-R criteria, with scores ranging from 0–63. It boasts high reliability (Cronbach’s α = 0.95, Guttman split-half = 0.91) and identifies two anxiety factors, subjective/panic, and somatic symptoms, explaining 58.04% of the variance. The threshold for anxiety is set between 13 and 14 points [27].

#### Functional constipation

According to the Rome III criteria^28^, functional constipation was defined as the presence of two or more of the following symptoms for at least three months, with onset at least six months earlier: straining, hard stools, a sensation of anorectal obstruction, incomplete evacuation, manual maneuvers to facilitate defecation, fewer than three weekly defecations, and rare loose stools without laxatives. Data were gathered from medical records, and interviews were conducted by data collectors.

### Data collection and research ethical considerations

The study, approved by the National Yang-Ming University’s IRB (YM107093EF), ensured full disclosure to participants about its purpose and procedures. Informed consent was obtained, with provisions for withdrawal without penalty. Privacy was safeguarded by using data exclusively for analysis, and questionnaires were anonymized and coded. We scheduled time to listen to the sounds of the participants' bowels in all four quadrants for one minute each, beginning from the right lower quadrant and proceeding clockwise before they ate breakfast and after waking up.

### Data analysis

Analysis in IBM SPSS Windows 24.0 involved descriptive statistics and statistical tests such as the χ^2^ test, Fisher’s exact test, and t-tests to examine the links between functional constipation and medication, lifestyle, and affective symptoms and to identify key predictors through logistic regression. When using a 2×2 contingency table to analyze the association between dichotomous variables and functional constipation, if the number of any of the four cells was < 5, the chi-square test was inappropriate, and we used Fisher’s exact test.

All multivariate logistic regression models included medical comorbidities and the chlorpromazine equivalent dose as control variables to determine the relationships between lifestyle, atypical antipsychotic drug use, affective symptoms, and constipation. In this study, multicollinearity was confirmed to be minimal, with a VIF below 1.5, indicating a significant association between anxiety and depressive symptoms (χ^2^ = 49.63, *p* < 0.001). A detailed examination of functional constipation required analyzing these symptoms separately due to their significant prevalence (15.13% with BDI ≥ 17 and BAI ≥ 14). Multiple logistic regressions, validated by the Hosmer–Lemeshow test, confirmed model fit (*p* > 0.05).

The sample size for the study was estimated using a two-tailed Z test in sample power software, taking depressive symptoms as an example. The odds ratio (OR) was set at 3.0, with a significance level (*p*) of less than 0.05 and a power of 0.80. The prevalence rate of constipation was set at 27.88% [7], and the prevalence rate of BDI ≥ 17 was set at 32% [29]. The required sample size was 183. Considering an estimated 30% rate of invalid questionnaires, this study’s target number of participants was 261. Post hoc power analysis confirmed high statistical power (0.89 for anxiety, 0.95 for depression) with a sample size of 271 participants.

## Results

### Participants’ characteristics

The study involved 275 participants, 271 of whom were included in the analysis. Tables 1 and 2 detail the relationships between demographics, lifestyle, clinical factors, and functional constipation. In our study, 57.56% of the participants who were administered one to two laxatives were categorized as having mild to moderate constipation or were using laxatives as a preventive strategy. Additionally, 4.06% of the patients who needed more than two laxatives at the same time were considered likely to suffer from severe chronic constipation or an acute worsening of their condition. Over 60% of respondents regularly used laxatives, 56.46% used anticholinergics, and 90.41% used atypical antipsychotics. Less than 24% consumed adequate fruit, and only 7.75% met vegetable intake recommendations. Most (81.5%) drank less than 2,000 cc of water; the prevalence of affective symptoms was greater than 25%.

**Table 1:** Comparison of demographic data, clinical factors, lifestyles and affective symptoms between patients with and without constipation (n = 271)

	Functional constipation				χ^2^	*p*
	No		Yes			
	*n*	%	*n*	%		
Variables	91	33.6	180	66.4		
Sex						
Female	32	35.2	104	57.8	12.363	< 0.001
Male	59	64.8	76	42.2		
Occupational training						
No	63	69.2	139	77.2	2.034	0.154
Yes	28	30.8	41	22.8		
Anti-cholinergic use						
No	45	49.5	73	40.6	1.945	0.163
Yes	46	50.5	107	59.4		
Laxative use						
No	62	68.1	42	23.3	51.293	< 0.001
Yes	29	31.9	138	76.7		
Medical comorbidity						
No	77	84.6	135	75.0	3.281	0.070
Yes	14	15.4	45	25.0		
Drinking > 3000 cc of water daily						
No	83	91.2	175	97.2	4.786	0.029
Yes	8	8.8	5	2.8		
Two servings of fruit daily						
No	66	72.5	141	78.3	1.129	0.288
Yes	25	27.5	39	21.7		
Three servings of vegetables daily						
No	85	93.4	165	91.7	0.256	0.613
Yes	6	6.6	15	8.3		
Smoker						
No	76	83.5	154	85.6	0.196	0.658
Yes	15	16.5	26	14.4		
Drinker						
No	85	93.4	168	93.3	0.001	0.982
Yes	6	6.6	12	6.7		
Beck Depression Inventory-II (BDI-II)						
BDI ≦ 16	78	85.7	124	68.9	9.016	0.003
BDI ≧ 17	13	14.3	56	31.1		
Beck Anxiety Inventory-II (BAI)						
BAI ≦ 13	78	85.7	120	66.7	11.142	0.001
BAI ≧ 14	13	14.3	60	33.3		

Variables	*M*	*SD*	*M*	*SD*	*t*	*p*
Age (years)	49.9	8.5	49.8	8.5	0.149	0.882
Body mass index (kg/m^2^)	24.6	4.3	25.1	4.8	0.797	0.426
Duration of disease (years)	22.1	10.5	23.9	10.7	–1.302	0.194
BPAQ	4.5	1.5	4.4	1.6	0.527	0.598
Chlorpromazine _(100 mg)_ equivalent	530.4	340.0	619.3	406.2	–1.900	0.059
Diazepam _(10 mg)_ equivalent	19.7	22.2	20.3	27.7	–0.187	0.851
Imipramine _(50 mg)_ equivalent	16.5	50.2	12.6	34.3	0.749	0.454
Bowel sounds						
Right lower quadrant	5.9	4.0	5.0	4.0	1.665	0.097
Right upper quadrant	6.1	3.8	5.4	3.8	1.377	0.170
Left upper quadrant	5.8	3.8	5.2	3.7	1.145	0.253
Left lower quadrant	5.4	4.0	5.0	4.0	0.649	0.517

BPAQ Baecke physical activity questionnaire

**Table 2:** Comparison of antipsychotic factors between patients with and without constipation (n = 271)

	Functional constipation				χ^2^	*p*
	No		Yes			
	*n*	%	*n*	%		
Variables	91	33.6	180	66.4		
Amisulpride						
No	87	95.6	173	96.1		1.000^a^
Yes	4	4.4	7	3.9		
Aripiprazole						
No	87	95.6	147	81.7		0.001^a^
Yes	4	4.4	33	18.3		
Clozapine						
No	64	70.3	137	76.1	1.055	0.304
Yes	27	29.7	43	23.9		
Olanzapine						
No	78	85.7	155	86.1	0.008	0.929
Yes	13	14.3	25	13.9		
Paliperidone						
No	86	94.5	172	95.6	0.146	0.702
Yes	5	5.5	8	4.4		
Quetiapine						
No	81	89.0	136	75.6	6.858	0.009
Yes	10	11.0	44	24.4		
Risperidone						
No	64	70.3	151	83.9	6.778	0.009
Yes	27	29.7	29	16.1		
Number of atypical antipsychotics						
0	10	11.0	10	5.6	3.447	0.178
1	70	76.9	139	77.2		
2	11	12.1	31	17.2		
Types of antipsychotics						
FGA only	12	13.2	11	6.1	4.381	0.112
SGA only	58	63.7	131	72.8		
FGA + SGA	21	23.1	38	21.1		
Low-potency antipsychotics						
No	79	86.8	157	87.2	0.009	0.924
Yes	12	13.2	23	12.8		
High-potency antipsychotics						
No	69	75.8	150	83.3	2.198	0.138
Yes	22	24.2	30	16.7		

^a^Fisher’s exact test

BDI Beck depression inventory II, BAI Beck anxiety inventory, FGA First‑generation antipsychotics, SGA Second‑generation antipsychotics

### Comparison of demographic data, medication factors, and lifestyle factors between participants with and without constipation

Based on the Rome III criteria, 180 participants (66.4%) had functional constipation. Chi-square or t-tests were used to examine the associations between independent variables and functional constipation (Tables 1 and 2). If the number of individuals was less than 5 after cross-tabulation, Fisher’s exact test was used to analyze the relationships between the variables and functional constipation. Table 1 illustrates significant associations between functional constipation and factors such as sex (χ^2^ = 12.363, *p* < 0.001), laxative use (χ^2^ = 51.293, *p* < 0.001), daily water intake of 3000 cc (χ^2^ = 4.786, *p* = 0.029), and affective symptoms (*p* = 0.029–0.001). Despite laxative use, 138 individuals continued to experience functional constipation. Notably, 29 participants received prophylactic laxatives to prevent constipation; 12 used clozapine, 4 used olanzapine, and 3 used quetiapine.

Age, BMI, disease duration, BPAQ score, medication equivalent, bowel sounds, employment status, anticholinergic use, comorbidities, daily intake of 2 fruit servings, three vegetable servings, and tobacco or cigarette use were not significantly associated with constipation.

### Comparison of atypical antipsychotic factors in patients with and without constipation

Table 2 displays the relationships between atypical antipsychotic use and functional constipation, with medications taken by fewer than 10 participants excluded from further analysis. The relationship between the use of three atypical antipsychotic drugs (aripiprazole, quetiapine, and risperidone) and functional constipation was significant.

Contrary to our expectations, clozapine (*p* = 0.304) and olanzapine (*p* = 0.929) did not have significant effects. Among the 70 participants who used clozapine, 38 were on clozapine monotherapy. Further analysis revealed that despite the lack of substantial differences, participants with constipation problems had an equivalent dose of antipsychotic medication of 522.92 mg (*SD* = 177.53), whereas those without constipation problems had an equivalent dose of 435.71 mg (*SD* = 169.19). In this study, 38 participants were administered olanzapine, 14 of whom received monotherapy. Further analysis of constipation revealed that the equivalent dosage of the antipsychotic olanzapine was 396.67 mg (*SD* = 202.13) among those with constipation and 300.00 mg (*SD* = 81.65) among those without constipation.

Our study included 42 participants who received treatment with two atypical antipsychotic medications. Notably, 19 of these participants followed a regimen including risperidone in combination with other antipsychotics. Specifically, the adjunct medications paired with risperidone were clozapine for 9 subjects, olanzapine for 8 subjects, and quetiapine for 2 subjects.

### Predictors of functional constipation in people with schizophrenia

We analyzed the significance of atypical antipsychotic and emotional symptoms as predictors of functional constipation adjusted for sex, anticholinergic and laxative use, water consumption, physical activity, laxative use, two servings of fruit daily, three servings of vegetables daily, drinking > 3000 cc of water daily, physical activity, medical comorbidity, and chlorpromazine equivalent dose.

#### Associations between sex, medication use, lifestyle factors, and the risk of functional constipation in people with schizophrenia

Table 3 outlines how sex, anticholinergic and laxative use, diet, water consumption, physical activity, medical comorbidity, and chlorpromazine equivalent dose predict constipation. Multivariate logistic regression revealed that female sex (*OR* = 2.977, *p* = 0.001), anticholinergic use (*OR* = 2.101, *p* = 0.017), and laxative use (*OR* = 6.734, *p* < 0.001) significantly increased constipation risk. Conversely, consuming more than 3,000 cc of water daily significantly reduced constipation risk (*OR* = 0.228, *p* = 0.028) as a protective factor. Notably, medical comorbidities and chlorpromazine equivalent doses did not have statistical significance; the observed trends suggest that higher doses of antipsychotics and chronic comorbidities are associated with increased risks of functional constipation.

**Table 3:** Multivariate logistic regression analysis of lifestyle factors associated with functional constipation (N = 271)

	B	SE	OR	95% CI for:	OR	*p*
Female	1.091	0.323	2.977	1.581	5.605	0.001
Anticholinergics	0.742	0.312	2.101	1.140	3.872	0.017
Laxatives	1.907	0.308	6.734	3.681	12.318	< 0.001
Two servings of fruit daily	–0.310	0.357	0.733	0.364	1.477	0.385
Three servings of vegetables daily	0.562	0.620	1.754	0.520	5.915	0.365
Drinking > 3,000 cc of water daily	–1.476	0.673	0.228	0.061	.854	0.028
Physical activity	–0.068	0.097	0.934	0.772	1.129	0.479
Medical comorbidity	0.710	0.402	2.035	0.926	4.472	0.077
Chlorpromazine equivalent dose	0.001	0.000	1.001	1.000	1.002	0.075
Constant	–1.437	0.616	0.238			0.020
Hosmer and Lemeshow Test: χ^2^ = 12.309, *df* = 8, *p* = .138						

#### Association between atypical antipsychotic drug use and the risk of functional constipation in people with schizophrenia

Table 4 Models 1–1, 2–1, and 3–1 include demographic data and lifestyle factors, clinical characteristics, and three atypical antipsychotics, namely, aripiprazole, quetiapine, and risperidone, with two control variables in the logistic regression models. Aripiprazole users had a 4.566-fold (*p* = 0.013), quetiapine users had a 2.396-fold (*p* = 0.046), and risperidone users had a 0.301-fold (*p* = 0.004) risk of constipation, suggesting that risperidone users were less likely to have functional constipation. Compared with other atypical antipsychotics in this study sample, aripiprazole users exhibited the highest risk of functional constipation among the three atypical antipsychotic drugs. The risk for aripiprazole users was 4.566 times greater than that for nonusers. The risk of functional constipation was 2.396 times greater for quetiapine users than for nonusers. Among the three atypical antipsychotic medications, risperidone had the lowest associated risk of functional constipation.

**Table 4:** *Atypical antipsychotics and emotional symptoms associated with functional constipation according to multivariate logistic regression* (*n* = 271)

	Model 1–1				Model 1–2				Model 1–3			
Predictors	OR	95% CI for:	OR	*p*	OR	95% CI for:	OR	*p*	OR	95% CI for:	OR	*p*
Constant	0.195			0.010	0.147			0.003	0.178			0.008
Female	3.031	1.582	5.807	0.001	2.836	1.470	5.470	0.002	2.787	1.436	5.409	0.002
Anti-Cholinergic	2.376	1.269	4.450	0.007	3.082	1.579	6.014	0.001	2.567	1.343	4.905	0.004
Laxatives	6.339	3.437	11.693	< 0.001	6.663	3.538	12.550	< 0.001	6.432	3.438	12.033	< 0.001
Two servings of fruit daily	0.802	0.393	1.636	0.544	0.811	0.390	1.690	0.577	0.752	.362	1.563	0.445
Three servings of vegetables daily	1.571	0.468	5.277	0.465	1.655	0.465	5.888	0.436	1.863	.532	6.527	0.331
Drinking > 3,000 cc of water daily	0.206	0.053	0.808	0.023	0.160	0.036	0.706	0.015	0.149	.034	.648	0.011
Physical activity	0.927	0.761	1.128	0.448	0.917	0.752	1.119	0.394	0.906	.740	1.108	0.337
Aripiprazole	4.566	1.370	15.221	0.013	4.992	1.459	17.080	0.010	5.360	1.562	18.396	0.008
BDI ≧ 17					3.868	1.683	8.886	0.001				
BAI ≧ 14									3.231	1.438	7.258	0.005
Hosmer and Lemeshow Test	χ^2^ = 8.123, *df* = 8, *p* = .422				χ^2^ = 7.841, *df* = 8, *p* = .449				χ^2^ = 9.663, *df* = 8, *p* = .289			

	Model 2–1				Model 2–2				Model 2–3			
Predictors	*OR*	95% CI for *OR*		*p*	*OR*	95% CI for *OR*		*p*	*OR*	95% CI for *OR*		*p*
Constant	0.209			0.013	0.163			0.005	0.207			0.014
Female	2.866	1.511	5.436	0.001	2.804	1.468	5.355	0.002	2.591	1.350	4.975	0.004
Anti-Cholinergic	2.094	1.128	3.888	0.019	2.604	1.356	5.000	0.004	2.161	1.151	4.057	0.016
Laxatives	7.162	3.866	13.268	< 0.001	7.419	3.942	13.963	< 0.001	7.231	3.864	13.534	< 0.001
Two servings of fruit daily	0.753	0.372	1.522	0.429	0.774	0.376	1.595	0.488	0.694	0.339	1.424	0.320
Three servings of vegetables daily	1.692	0.490	5.843	0.406	1.785	0.489	6.508	0.380	1.989	0.556	7.118	0.290
Drinking > 3,000 cc of water daily	0.246	0.064	0.942	0.041	0.193	0.044	0.845	0.029	0.187	0.044	0.797	0.023
Physical activity	0.944	0.777	1.145	0.557	0.936	0.771	1.138	0.509	0.928	0.762	1.130	0.455
Quetiapine	2.396	1.018	5.639	0.046	2.272	0.947	5.453	0.066	2.263	0.945	5.420	0.067
BDI ≧ 17					3.520	1.553	7.979	0.003				
BAI ≧ 14									2.787	1.256	6.184	0.012
Hosmer and Lemeshow Test	χ^2^ = 7.831, *df* = 8, *p* = .450				χ^2^ = 12.559, *df* = 8, *p* = .128				χ^2^ = 11.637, *df* = 8, *p* = .168			

	Model 3–1				Model 3–2				Model 3–3			
Predictors	*OR*	95% CI for *OR*		*p*	*OR*	95% CI for *OR*		*p*	*OR*	95% CI for *OR*		*p*
Constant	0.227			.019	0.163			.006	0.211			.016
Female	3.053	1.597	5.838	.001	2.985	1.543	5.772	.001	2.781	1.437	5.381	.002
Anti-Cholinergic	3.145	1.560	6.341	.001	4.207	2.000	8.850	< .001	3.266	1.592	6.700	.001
Laxatives	7.337	3.918	13.740	< .001	7.660	4.006	14.647	< .001	7.403	3.907	14.027	< .001
Two servings of fruit daily	0.913	0.438	1.900	.807	0.954	0.447	2.035	.903	0.825	0.391	1.740	.613
Three servings of vegetables daily	1.661	0.484	5.706	.420	1.752	0.475	6.455	.399	1.901	0.531	6.801	.324
Drinking > 3,000 cc of water daily	0.242	0.064	0.905	.035	0.196	0.045	0.853	.030	0.194	0.047	0.801	.023
Physical activity	0.946	0.779	1.149	.576	0.947	0.779	1.153	.590	0.936	0.769	1.140	.513
Risperidone	0.301	0.133	0.684	.004	0.258	0.109	0.611	.002	0.301	0.130	0.699	.005
BDI ≧ 17					3.949	1.742	8.955	.001				
BAI ≧ 14									2.821	1.277	6.229	.010
Hosmer and Lemeshow Test	χ^2^ = 10.400, *df* = 8, *p* = .238				χ^2^ = 5.482, *df* = 8, *p* = .705				χ^2^ = 8.846, *df* = 8, *p* = .355			

BDI Beck depression inventory II, BAI Beck anxiety inventory, Control variables: medical comorbidity, chlorpromazine equivalent dose

#### Associations between anxiety, depressive symptoms, and the risk of constipation in patients with schizophrenia

Table4 shows the demographic data, lifestyle factors, clinical characteristics, and use of three atypical antipsychotics with two control variables. Models 1–2, 2–2, and 3–2 also considered depressive symptoms (BDI ≥ 17) as predictors, along with the effects of aripiprazole, quetiapine, and risperidone. After adjusting for factors such as sex, anticholinergic and laxative use, diet, water consumption, physical activity, medical comorbidity, chlorpromazine equivalent dose, and atypical antipsychotic use, a BDI score ≥ 17 significantly increased constipation risk by 3.520–3.949 times, highlighting depressive symptoms as a risk factor even when multiple variables were considered.

Like the data analysis and processing methods for BDI ≥ 17 as a predictor, Table 4 models 1–3, 2–3, and 3–3 present the results for BAI ≥ 14 as a predictor. According to the multivariate analysis, a participant with a high BAI score (≥ 14) had a greater risk than a participant with a low BAI score (*OR* = 2.787–3.231).

All the predictive models were fitted by the Hosmer‒Lemeshow goodness-of-fit test, with χ^2^ values < 13 and all *p* > 0.05, showing that the model fit the data well.

After adjusting for vegetable and fruit fiber intake, physical activity, and laxative use, female sex, BAI score ≥ 14, and BDI score ≥ 17 emerged as risk factors for functional constipation. Conversely, consuming 3000 cc of water daily and using risperidone are associated with a lower risk of constipation. While anticholinergic medication use initially showed no significant association with constipation, it was identified as a risk factor when considering the combined effects of medications, lifestyle, and emotional symptoms.

#### Atypical antipsychotics and emotional symptoms associated with functional constipation and anticholinergic subgroup analysis

Given the established link between anticholinergic medications and functional constipation, as shown by previous studies, and the frequent use of anticholinergic drugs among patients with schizophrenia, as indicated in some research, we conducted an anticholinergic subgroup analysis to clarify the relationships between these medications and the predictors of affective symptoms and constipation. As shown in Table5, patients with schizophrenia who were exposed to the following antipsychotic medications had a significantly greater risk of constipation than nonusers after controlling for sex, laxative use, two servings of fruit daily, three servings of vegetables daily, drinking more than 3000 cc of water daily, physical activity, medical comorbidity, and chlorpromazine equivalent dose.

**Table 5:** Atypical antipsychotics and emotional symptoms associated with functional constipation and anticholinergic subgroup analysis

	Anti-Cholinergics							
	Non-user (n = 118)				User (n = 153)			
	*B*	*SE*	*OR*	*p*	*B*	*SE*	*OR*	*p*
Model 1a Aripiprazole	1.453	0.789	4.278	.066	1.699	1.270	5.466	.181
BDI≧17	1.331	0.547	3.785	.015	1.304	0.705	3.684	.064
Model 1b Quetiapine	-0.066	0.666	0.936	.921	1.705	0.719	5.499	.018
BDI≧17	1.330	0.538	3.781	.013	1.205	0.709	3.336	.089
Model 1c Risperidone	-2.627	1.354	0.086	.052	-1.237	0.496	0.290	.013
BDI≧17	1.350	0.551	3.857	.014	1.507	0.727	4.512	.038

	Anti-Cholinergics							
	Non-user (n = 118)				User (n = 153)			
	*B*	*SE*	*OR*	*p*	*B*	*SE*	*OR*	*p*
Model 2a Aripiprazole	1.522	0.777	4.582	.050	1.459	1.325	4.303	.271
BAI≧14	0.452	0.558	1.572	.418	2.299	0.810	9.964	.005
Model 2b Quetiapine	0.034	0.626	1.034	.957	1.361	0.744	3.898	.068
BAI≧14	0.306	0.552	1.359	.579	2.083	0.822	8.031	.011
Model 2c Risperidone	-2.590	1.358	0.075	.056	-0.980	0.491	0.375	.046
BAI≧14	0.302	0.561	1.353	.590	2.261	0.809	9.595	.005

Control variables: sex, laxatives, two servings of fruit daily, three servings of vegetable daily, drink > 3,000 cc of water daily, and physical activity, medical comorbidity and chlorpromazine equivalent dose

BDI Beck depression inventory II, BAI Beck anxiety inventory, Control Variables: medical comorbidity, chlorpromazine equivalent dose

There was a significant association for quetiapine users, with a higher odds ratio (*OR*) of 5.499 (*p* = 0.018) when considering anticholinergics. For risperidone users, there was a significant negative association, with an OR of 0.290 (*p* = 0.013) when anticholinergics were used. No significant associations were found with aripiprazole.

When considering depressive symptoms, there was a significant association with greater odds of having a BDI score ≥ 17 for nonusers of anticholinergics, with ORs of 3.785 (*p* = 0.015) for aripiprazole users and 3.781 (*p* = 0.013) for quetiapine users. For risperidone, the association was significant for nonusers (*OR* = 3.857, *p* = 0.014) and users (*OR* = 4.512, *p* = 0.038) of anticholinergics. The results revealed significant associations for BAI ≥ 14 between aripiprazole, quetiapine, and risperidone users with *ORs* of 9.664 (*p* = 0.005), 8.031 (*p* = 0.011) and 9.595 (*p* = 0.005), respectively.

Subgroup analysis revealed that a BDI score ≥ 17 is a risk factor for functional constipation for nonusers of anticholinergics. Among users of anticholinergics, BAI ≥ 14 and BDI ≥ 17 were significantly associated with functional constipation.

## Discussion

This study in northern Taiwan assessed factors such as diet, hydration, physical activity, and medication use affecting functional constipation in schizophrenia patients at rehabilitation centers, revealing a 66.4% prevalence rate, which was slightly higher than that in eastern Taiwan (59.8%) [5]. This difference may be due to the sample’s sex composition and anticholinergic use, with Chang and Chen (2021) reporting a male majority (64.3%) and 32.8% anticholinergic use [5]. In contrast to more than 90% of participants in previous studies who reported regular exercise and high-fiber diets, approximately 50% of participants in this study were female, 56.46% were on anticholinergics, and less than 25% had a high-fiber diet. Past research indicates that females [3, 30] and anticholinergic users are at a greater risk of constipation [5], while high-fiber diets and regular exercise typically reduce constipation risk [31]. However, our findings suggest a minimal impact of physical activity on constipation, possibly due to lower activity levels than in previous studies [9, 10]. Low vegetable/fruit consumption and high anticholinergic and atypical antipsychotic use were identified as risk factors [32].

We evaluated factors contributing to functional constipation, including dietary intake of water, vegetables, and fruits, physical activity, sex-specific adjustments, and anticholinergics and laxatives. Research from India showed that individuals who consumed less than 2,400 cc of water daily and who led a sedentary lifestyle were more susceptible to constipation^33^. Similarly, a study from Turkey revealed that elderly individuals without constipation consumed an average of 2200 cc of water daily, exceeding the intake of those with constipation who consumed less than 2000 cc^34^. After adjusting for eight controls, our study indicated that consuming more than 3000 cc of water daily reduced constipation risk. However, excessive water intake can lead to water intoxication and hyponatremia, which dilutes blood electrolytes, notably sodium, potentially causing dizziness, nausea, confusion, seizures, and respiratory distress. Standard recommendations suggest that adequately hydrated women and men consume approximately 2.7 L (91 oz) and 3.7 L (125 oz) of water daily, respectively, with 80% from beverages and 20% from food^35^. Hence, psychiatric professionals recommend that patients drink water frequently and slowly throughout the day.

While previous research linked constipation to anticholinergics or laxatives [16], our study revealed no direct impact of anticholinergics on constipation. However, after adjusting for other variables, including eight controls or atypical antipsychotic use, anticholinergics emerged as a risk factor for functional constipation, consistent with past findings. Anticholinergic drugs inhibit acetylcholine, affecting the central, autonomic nervous, and digestive systems. However, these drugs might not directly cause constipation but may increase the risk when combined with antipsychotics [5, 36].

A study by Indian scholars [37] showed that clozapine users have a 56% increased risk of constipation, which is significantly greater than that of nonusers, whereas clozapine and olanzapine did not have a significant effect on constipation in our study. A possible explanation might be that when physicians prescribe clozapine and olanzapine, they may limit the equivalent dose of antipsychotic drugs to < 400 mg [38] or combine them with other second-generation antipsychotics, such as risperidone or paliperidone. In this study, 61.26% of the participants used laxatives, among whom 29 individuals (10.70%) received routine prophylactic administration without having functional constipation rather than using laxatives as needed (prn) when constipation occurred. Previous research on the link between antipsychotic use and constipation risk has primarily examined clozapine-induced constipation, identifying risk factors such as female sex, advanced age, anticholinergic medication use, obesity, dietary fiber intake, and drug dosage [20, 39]. When prescribing clozapine, medical teams typically advise minimizing medications with anticholinergic effects, managing body weight, and taking additional precautions for females to reduce constipation risk [40, 41]. In contrast to aripiprazole and quetiapine users, risperidone users have a lower risk of constipation. Compared with other atypical antipsychotics, risperidone has a lower anti-motility effect [32, 42], possibly explaining the lower risk for constipation in risperidone users.

The prevalence of depressive and anxiety symptoms among stable-phase schizophrenia patients was 25.46% and 26.94%, respectively, and these conditions were linked to reduced physical activity levels [43]. Affective symptoms, which impede physical activity, were associated with a 3.520– 3.949-fold and a 2.787– 3.231-fold increased risk of constipation in participants with depression and anxiety, respectively [44]. Depression often leads to decreased physical activity, affecting gastrointestinal motility and bowel movements. Antidepressants with anticholinergic effects can further reduce peristalsis, heightening the risk of constipation [42]. In the United States, adults with depressive symptoms face a 2.76-fold greater risk of constipation [45]. Anxiety can also worsen this condition by activating the sympathetic nervous system, which diminishes gastrointestinal motility [46, 47]. Additionally, disruptions in the gut-brain axis may affect gastrointestinal motility in individuals with anxiety [48].

Although physical activity, vegetable and fruit intake, and clozapine use did not significantly impact this study, their importance remains. Contrary to previous beliefs that 2000 cc of daily water is sufficient for constipation prevention, this research suggested that patients with schizophrenia may require an additional 1000 cc to avoid constipation, considering various complex factors. Psychiatric professionals must be cautious when advising patients to increase water intake, recommending “a small amount, many times, and slowly”.

Subgroup analysis indicated that a BDI score ≥ 17 is a risk factor for functional constipation in nonusers of anticholinergic drugs, while a BAI score ≥ 14 and a BDI score ≥ 17 are associated with functional constipation in users. These findings suggest a link between depressive and anxiety symptoms and functional constipation [49], particularly in patients using anticholinergic medications [50], which can affect bowel movements and exacerbate constipation [20]. This study highlights the importance of considering mental health status, especially depressive and anxiety symptoms, in treating patients taking anticholinergic medications.

Future studies should further investigate the longitudinal impacts of psychological factors and anticholinergic drug use on functional constipation and develop comprehensive treatment strategies to improve constipation in patients with schizophrenia. Healthcare providers must remain vigilant in supporting and treating these patients.

### Limitations

We analyzed constipation risk factors from various perspectives, but there are several limitations. 1. Cross-sectional studies only describe relationships without establishing causality. 2. Among the participants, 61.62% used laxatives; however, the analysis did not account for medication dosage, efficacy, duration of use, or differences within the diagnostic category, such as subtypes and symptom severity, potentially underestimating the prevalence of functional constipation. 3. Interactions between multiple medications were not examined. 4. Water, vegetable, and fruit intake data were self-reported, necessitating more objective measures. 5. Medical comorbidities are chronic diseases requiring treatment to maintain stability. This definition does not include undiagnosed or untreated comorbid conditions; thus, we did not account for potential comorbidities. Furthermore, the severity and number of comorbidities were not included in the scope of this research. 6. The results for the independent variables in the analysis may have been influenced by potential bias from clinicians who, aware of the significant risk of constipation associated with clozapine and olanzapine, implemented various strategies to mitigate this risk. 7. We collected data solely on anticholinergics and did not compute the equivalents for benztropine. Medications with antipsychotic properties that exhibit anticholinergic effects were not categorized as anticholinergics. Despite these limitations, the results can still serve as a clinical reference.

## Conclusion

We examined the impact of lifestyle, atypical antipsychotic use, and affective symptoms on constipation in schizophrenia patients. The findings show that female sex and anxiety or depressive symptoms notably heighten constipation risk. While quetiapine and aripiprazole increase this risk, risperidone may reduce it. A high water intake (over 3000 cc) also reduces constipation. Anxiety and depression remained significant factors even when controlling for sex, medication side effects, diet, and exercise.
