# Correction: Associations between constipation risk and lifestyle, medication use, and affective symptoms in patients with schizophrenia: a multicenter cross-sectional study

**DOI:** 10.1007/s00127-024-02743-w

**Published:** 2024-08-06

**Authors:** Che-Yu Chiang, Su-Chen Lo, Jason W. Beckstead, Chiu-Yueh Yang

**Affiliations:** 1 Department of Family and Community Medicine, Cathay General Hospital, Taipei, Taiwan; 2 National Yang Ming Chiao Tung University, Taipei, Taiwan; 3 College of Public Health, University of South Florida, Tampa, FL USA

**Correction to: Social Psychiatry and Psychiatric Epidemiology**

10.1007/s00127-024-02729-8

In this article the statement in the Funding information section was incorrectly given as ‘Open Access funding enabled and organized by National Yang Ming Chiao Tung University’ and should have read ‘Open Access funding is enabled and organized by National Yang Ming Chiao Tung University. The study was supported by the National Science and Technology Council, Taiwan (NSTC107-2314-B-010-055)’.

The original article has been corrected.

